# The Effect of Cardiac Rehabilitation on Lipid Levels in Patients with Coronary Heart Disease. A Systematic Review and Meta-Analysis

**DOI:** 10.5334/gh.1170

**Published:** 2022-11-29

**Authors:** Gang Wu, Yemei Hu, Kun Ding, Xuedong Li, Jun Li, Zhuo Shang

**Affiliations:** 1 Department of Cardiology, Bengbu second people’s hospital, Bengbu, Anhui province, 233000, PR, China; 2 Bengbu second people’s hospital, No. 302 of Yan’ an Road, Bengbu, Anhui province, 233000, PR, China

**Keywords:** Coronary disease, coronary disease rehabilitation, lipid profile, randomized controlled trials

## Abstract

**Background:**

Cardiac rehabilitation (CR) is a multidisciplinary medical program. Most studies have emphasized the effect of exercise-based CR in lowering lipid levels; however, the effect of CR as a comprehensive program on lipid levels remains unclear.

**Methods:**

Electronic database were searched up to 2022. Randomized controlled trials with lipid profile indicators were included. Standardized mean differences (SMDs) and 95% CIs were used to evaluate the effect size. Begg’s funnel plot and Egger’s linear regression test were used to assess publication bias.

**Results:**

CR remarkably reduced low-density lipoprotein cholesterol (LDL-C) levels (SMD = –0.23; 95%CI: [–0.38, –0.08]; P < 0.001), triglyceride (TG) levels (SMD = –0.17; 95%CI: [–0.28, –0.06]; P < 0.001), and total cholesterol (TC) levels (SMD = –0.30; 95%CI: [–0.43, –0.16]; P < 0.001) and increased high-density lipoprotein cholesterol (HDL-C) levels (SMD = 0.19; 95%CI: [0.10, 0.29]; P < 0.001).

**Conclusions:**

CR reduce TC, TG, and LDL-C levels while improving HDL-C levels. CR should be promoted and more trials should be conducted for long-term CR.

## Introduction

Coronary heart disease (CHD) is a type of heart disease characterized by progressive coronary atherosclerotic lesions and the accumulation of fatty deposits along the inner layer of the coronary arteries, resulting in narrowing or blockage of coronary arteries and myocardial ischemia. CHD is a major component of cardiovascular disease and the leading cause of morbidity and mortality in the developed world [1,2]. Patients diagnosed with CHD are at increased risk of premature death, myocardial infarction, and readmission to hospital. CHD is an important public health concern and economic burden worldwide [3]. According to clinical guidelines, secondary prevention strategies are crucial after diagnosis because controlling risk factors is the key to recurrence management [4].

Cardiac rehabilitation (CR) is an integration of multiple coordinated, intentional interventions that could offer a comprehensive platform for the administration of secondary prevention measures, such as evidence-based pharmacotherapy, cardiac risk factor management, nutritional counselling, patient education, and physical activity training [5,6].

The proper control of risk factors, such as blood pressure and lipid levels, play an important role in decelerating disease progression and preventing recurrence and complications. Several meta-analyses have shown that exercise-based CR lowers blood lipid levels and blood pressure [7,8]. However, CR is an integrated and comprehensive program; therefore, we have updated and supplemented the data with the aim to measure the effect of multiple modalities of CR on lipids.

## Methods

We performed this systematic review and meta-analysis of trials to investigate CR interventions for patients with coronary heart disease (CHD) according to the Preferred Reporting Items for Systematic Reviews and Meta-Analyses (PRISMA) statement.

### Literature search strategy

Relevant studies were identified through a systematic search of the following electronic databases: MEDLINE, PubMed, EMBASE (Ovid), Web of Science, Scopus, and Cochrane Central Register of Controlled Trials up to 2022. The following Medical Subject Headings (MeSH) terms: ‘cardiac rehabilitation’ or ‘lifestyle change’ or ‘exercise training’ or ‘prevention program’ and ‘CHD’ or ‘CAD’ or ‘AMI’ or ‘ACS’ and ‘lipid profile’ or ‘HDL-C’ or ‘LDL-C’ or ‘TC’ or ‘TG’ were applied in our search strategy. We manually reviewed relevant review articles, reference lists of published trials, and conference abstracts (American Society of Clinical Oncology [ASCO], Annual Meetings, and the European Cancer Conference [ECCO]) for potentially eligible studies.

### Inclusion and exclusion criteria for study collection

Studies were included if they met the following criteria: 1) controlled trials of cardiac rehabilitation (life modification, exercise training, etc.) regardless of whether it included allocation concealment or blinding; 2) adult (age ≥ 18 years) patients with coronary artery diseases (CAD) or CHD; 3) intervention group involved patients undergoing cardiac rehabilitation and control group included patients undergoing usual care or medication; and 4) measurement indicators were lipid profiles such as LDL-C, HDL-C, TC, TG. Studies were excluded if they conformed to the following criteria: 1) articles were not in English; 2) studies with a control group of home-based or community-based cardiac rehabilitation; and 3) studies with insufficient information or data. Two investigators independently checked whether each study met the inclusion criteria, and discrepancies were resolved by a third reviewer.

### Data extraction

Two researchers independently extracted basic information from the included studies (title, first author, publication year, author information, and document source) and characteristics of study patients (sex, age, country, sample size, and interventions) for both the experimental and control groups and obtained outcome measurements. Any inconsistencies were resolved through consensus.

### Quality assessment

Study quality was assessed using the Newcastle-Ottawa Scale (NOS). The total scores ranged from 0 to 9 points; trials considered ‘high quality’ scored higher than six. Two reviewers assessed the quality of the studies separately, and discrepancies were assessed by a third researcher.

### Statistical analysis

Statistical analyses were performed using Stata statistical software (version 13.0; Stata Corp., College Station, TX, USA). SMD and 95% CIs were used to evaluate the effect size. The Q test and I^2^ analysis, with a significance level of <0.1 were employed to assess the heterogeneity between studies. Meta-regression analysis was used to analyze the source of heterogeneity. Begg’s funnel plot and Egger’s linear regression test were used to investigate the possibility of publication bias. Differences were considered statistically significant at P < 0.05.

## Results

### Literature selection

**Figure 1:**
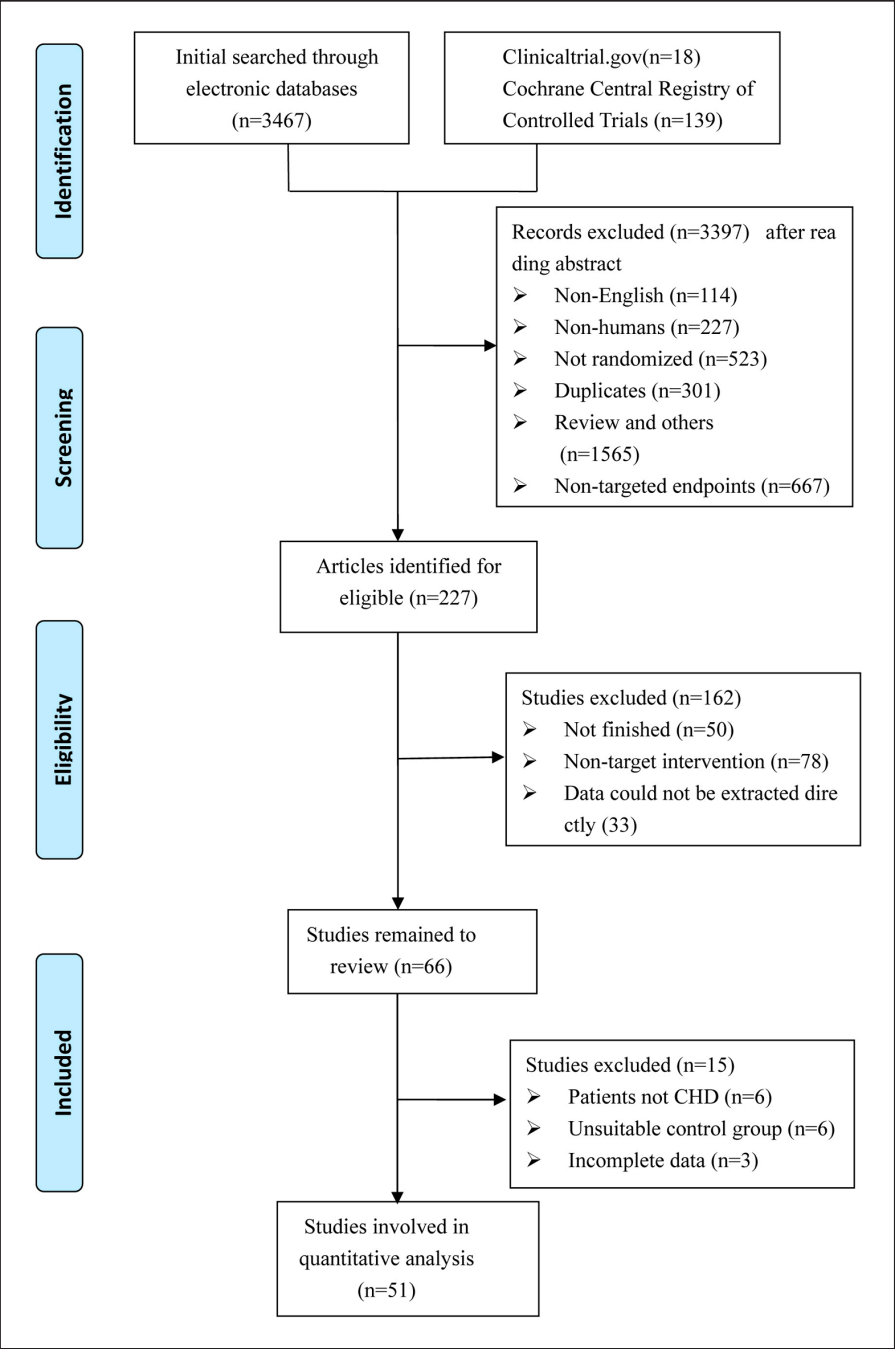
Diagram of including studies selection procedures.

In total, 3,624 potentially relevant articles were identified through an online search. After careful examination of titles and abstracts, 3,397 were excluded; 1,565 publications were excluded as review articles and other ineligible types of references, 114 were non-English language articles, 227 included experiments on non-human species, 301 articles were duplicates, and 667 had non-targeted endpoints (Figure 1). The remaining 227 articles were retrieved for more detailed assessment. After screening the full text, 162 studies were further excluded because of incomplete data (n = 50), non-target interventions (n = 78), data that could not be extracted directly (n = 33), not including non-CHD patients (n = 6), unsuitable control groups (n = 6), and incomplete data (n = 3). Ultimately, 51 studies were included in the meta-analysis [9,10,11,12,13,14,15,16,17,18,19,20,21,22,23,24,25,26,27,28,29,30,31,32,33,34,35,36,37,38,39,40,41,42,43,44,45,46,47,48,49,50,51,52,53,54,55,56,57,58,59].

### Characteristics of included studies

Fifty-one eligible studies with 10,286 patients were included, all of which were published between 1979 and 2022. The sample sizes ranged from 15 to 903 patients (median = 63). The age of all included patients ranged from 51 to 77.3 years old (median = 58.6); three studies had a three-arm parallel design, while forty-eight studies had a two-arm parallel design (CR and comparison). In Wosornu et al.’s [15] study, participants were assigned to three groups (control group, aerobic group, and strength group), which included people in CR, case-managed, and community-based groups. In a study by Volaklis et al., patients were grouped into land exercise, water exercise, and control. Nine studies were performed in the USA, five in the UK, six in Australia, four in Canada, three in Norway, three in HK, four in Sweden, two in Germany, two in Italy, two in Greece, fifteen studies from other countries. Forty-five studies collected TC measurements. Thirty-seven studies provided information on HDL-C as an endpoint, and forty-three studies used.

LDL-C as the endpoint. Thirty-eight studies used TG levels as the endpoint (Table 1).

**Table 1:** Characteristics of Including Studies.


AUTHOR	COUNTRY	TYPE OF DISEASE	NO. OF PATIENT		AGE MEAN (SD)		GENDER(M/F)		INTERVENTION	NOS SCALE
		
			T	C	T	C	T	C		

Kallio 1979	Finland	AMI	183	187	54.4	54.1	151/37	150/37	Health education Exercise training	6

Ornish 1990	USA	CAD	22	19	56.1(7.5)	59.8(9.1)	21/1	15/4	Lifestyle modification	8

Schuler 1992	Germany	CAD	56	57	52.8(5.8)	54.2(7.7)	NA	NA	Lifestyle modification Exercise training	8

Debusk 1994	USA	AMI	293	292	57(8)	57(8)	230/63	231/61	Lifestyle modification Exercise training	7

Fletcher 1994	USA	CAD	16	19	62(8)	63(7)	16/0	19/0	Exercise training	6

Haskwell 1994	USA	CAD	119	127	58.3(9.2)	56.2(8.2)	99/20	117/10	Lifestyle modification	7

Wosornu^a^ 1996	UK	CAD	27	27	56.5(8.9)	56.6(7.0)	27/0	27/0	Aerobic exercise training	8

Wosornu^b^ 1996	UK	CAD	27	27	59.2(6.4)	56.6(7.0)	27/0	27/0	Strength exercise training	8

Gallacher 1997	UK	CAD	198	209	NA	NA	NA	NA	Education (Stress management)	8

Niebauer 1997	UK	CAD	56	57	NA	NA	56/0	57/0	Lifestyle modification Exercise training	9

Ornish 1998	USA	ACS	20	15	57.4(6.4)	61.8(7.5)	20/0	12/3	Lifestyle modification	9

Carlsson^a^ 1998	Sweden	CAD	75	67	62.2(5.8)	61.7(6.0)	58/17	51/16	Lifestyle modification Exercise training	8

Carlsson^b^ 1998	Sweden	CAD	31	32	62.7(4.8)	59.8(4.8)	26/5	27/5	Lifestyle modification Exercise training	8

Bang 1999	Sweden	CAD	46	41	53(7)	53(7)	37/9	36/5	Lifestyle modification	8

Toobert 2000	USA	CHD	14	10	64(10)	63(11)	NA	NA	Lifestyle modification	8

Belardinelli 2001	Italy and USA	CAD	59	59	53(11)	59(10)	49/10	50/9	Exercise training	8

Gordon ^a^ 2002	USA	CAD	45	52	60(9)	61(10)	34/11	38/14	Lifestyle modification Education	8

Gordon ^b^ 2002	USA	CAD	45	45	60(9)	60(9)	34/11	35/10	Lifestyle modification Education	8

Vale 2002	Australia	CAD	107	112	61.5(10.3)	60.6(9.5)	80/27	84/28	Education Exercise training	7

Yu 2003	HK	CHD	72	40	62.3(11.2)	61.2(10.2)	59/13	30/10	Exercise training	6

The Vestfold Heartcare Study Group 2003	Norway	CHD	98	99	54(8)	55(8)	79/19	83/16	Lifestyle modification	8

Lear 2003	Canada	CAD	151	151	64.8(8.8)	63.4(10.2)	125/26	124/27	Lifestyle modification	8

Vale 2003	Australia	CHD	398	394	58.6(10.6)	58.3(10.3)	313/85	297/97	Education Exercise training	7

Vona 2004	Italy	AMI	28	24	56(6)	57(8)	21/7	19/5	Exercise training	7

Volaklis^a^ 2007	Greece	CAD; MI	12	10	53(4)	51(3)	NA	NA	Exercise training	7

Volaklis^b^ 2007	Greece	CAD; MI	12	10	58 (3)	51(3)	NA	NA	Exercise training	7

Zutz 2007	Canada	CAD	8	7	58(4)	59(12)	7/1	5/2	Lifestyle modification	7

Naser 2008	Iran	CHD	50	50	54.8	53.2	45/5	39/11	Lifestyle modification	7

Seki 2008	Japan	CHD; ACS; MI	14	10	64(10)	63(11)	NA	NA	Exercise training	7

Balen 2008	Croatia	AMI	30	30	59 (9)	61 (10)	21/9	23/7	Exercise training	7

Murphy 2009	Ireland	MI	444	459	68.5(9.3)	66.5(9.9)	311/133	320/139	Lifestyle modification Education	7

Zetta 2011	UK	CAD	109	109	65.9(10.0)	64.8(10)	71/38	78/31	Lifestyle modification Education	7

Munk 2011	Norway	CAD	18	18	59.5(10)	60.7(9)	16/2	14/4	Exercise training	7

Moholdt 2012	Norway	MI	59	30	57.5(9.3)	56.7(10.4)	49/10	25/5	Exercise training	7

Oerkild 2012	Denmark	MI	19	21	77.3(6.0)	76.5(7.7)	12/7	11/10	Lifestyle modification Education	8

Vernooij 2012	Netherland	CAD	164	166	60.7(7.8)	59.2(8.9)	128/36	118/48	Lifestyle modification Education	8

Luk 2012	HK	CAD	32	32	67.7(9.0)	66.6(7.9)	24/8	24/8	Exercise training	7

Lear 2014	Canada	CVD	38	40	59.2(10.7)	58.6(9)	34/4	32/8	Lifestyle modification Exercise training Education by internet	8

Raghuram 2014	India	CAD	129	121	53.3(6.4)	52.6(6.9)	NA	NA	Yoga training	8

Chow 2015	Australia	CHD	352	358	57.9(9.1)	57.3(9.3)	287/65	295/63	Lifestyle modification	7

Hassan 2016	Egypt	CAD	30	30	NA	NA	21/9	20/10	Lifestyle modification Education	8

Johnston 2016	Sweden	MI	86	80	56.8(8.0)	58.4(8.6)	71/15	63/17	Lifestyle modification Education	7

Tamburús 2016	Brazil	CAD	15	17	NA	NA	NA	NA	Exercise training	8

Lin 2017	HK	CAD	144	144	75.3(7.5)	74.3(7.5)	94/50	97/47	Education	7

Park 2017	South Korea	AMI	32	32	57(12)	55(10)	29/3	27/5	Education	7

Dorje 2019	Australia	MI; unstable or stable angina	156	156	59.1(9.4)	61.9(8.7)	128/28	126/30	Lifestyle modification Education by Wechat	8

Maddison 2019	Australia; New Zealand	CHD	82	80	61(13.2)	61.5(12.2)	69/13	70/10	Lifestyle modification Education	8

Santo 2019	Australia	CHD	107	56	58.4(9.0)	56.8 (8.6)	93/14	50/6	Education	7

Wienbergen 2019	Germany	MI	143	138	56.5(9.1)	56.4 (10.3)	117/36	112/26	Education telephone visits and telemetric risk factor control	8

Chaves 2019	Canada	MI	12	11	60.7(13.3)	60.6 (8.4)	12/0	4/7	Lifestyle modification Education	8

Zheng 2019	China	AMI	411	411	56.2(9.3)	56.6 (9.7)	353/58	353/58	Lifestyle modification Education by text messages	8

Campo 2020	Italy	ACS	118	117	76(6)	76.6 (5.2)	92/26	89/28	Lifestyle modification Education	8

Uddin 2020	USA	CHD	71	71	54(6)	55(6)	66/5	63/8	Lifestyle modification Education	8

Yudi 2021	Australia	ACS	83	85	56.8(9.9)	56.2 (10.2)	71/12	69/16	Lifestyle modification Education by smartphone	7

Dalli Peydro 2022	Spain	ACS	31	28	57.5(9.0)	54.7(9.9)	27/4	27/1	Lifestyle modification Education by smartphone	7


ACS: acute coronary syndrome; AMI: acute myocardial infarction; CAD: coronary atherosclerotic heart disease; CHD: coronary heart disease; CVD: coronary vascular disease; MI: myocardial infarction; NA: not available; NOS: Newcastle–Ottawa Scale.

### Meta-analysis results

#### TC levels

**Figure 2:**
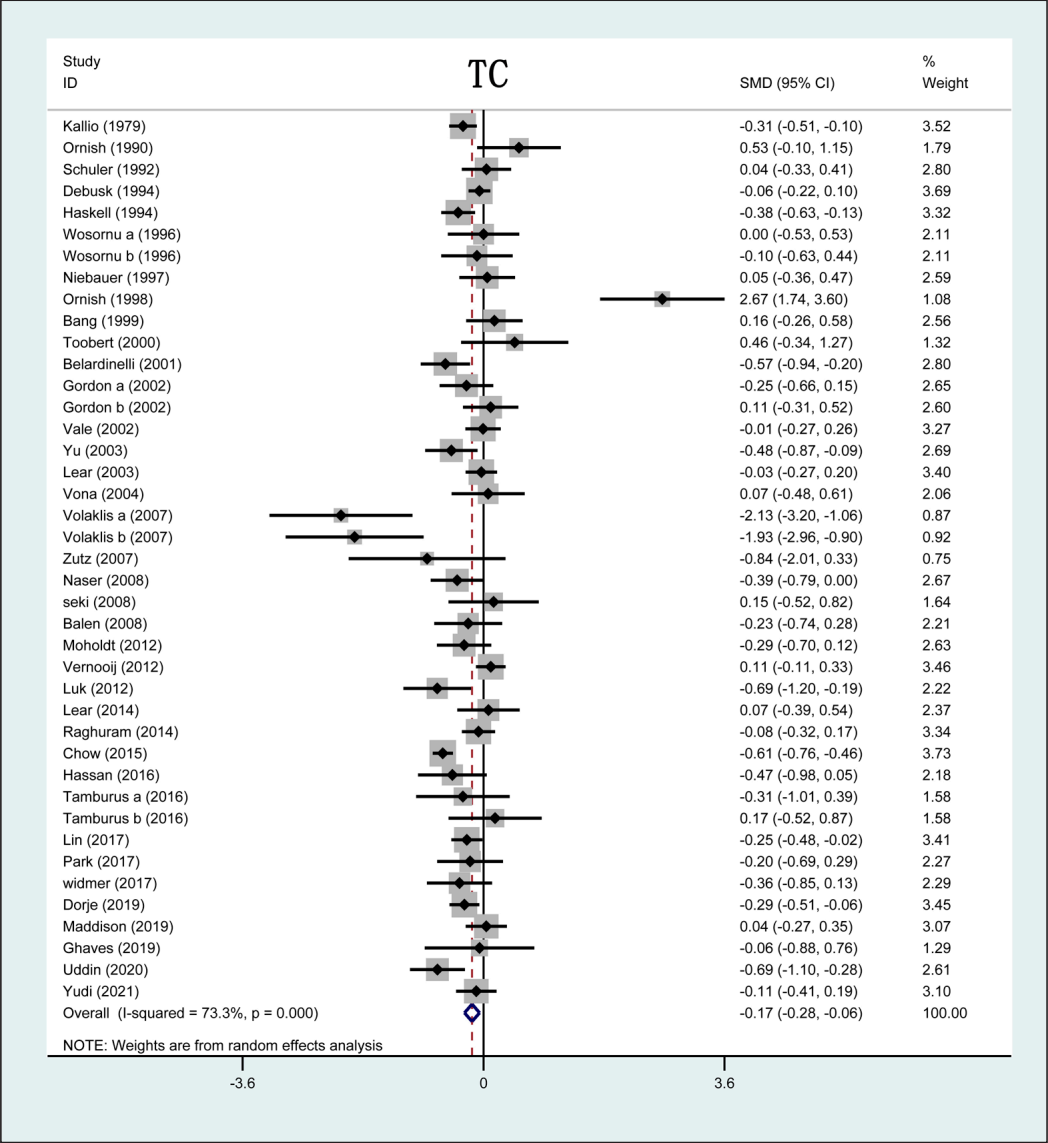
Forrest plot of TC levels after CR treatment. TC: total cholesterol; CR: cardiac rehabilitation; SMD: standardized mean difference.

Forty-four out of 51 studies included in our analysis reported on TC levels after CR. The forest plot showed that ten studies had significantly lower TC serum levels in the intervention group. The results of the studies reported that CR could significantly reduce TC serum levels compared with control groups (SMD = –0.30; 95%CI: [–0.43, –0.16]; P < 0.001, Figure 2).

#### HDL-C levels

**Figure 3:**
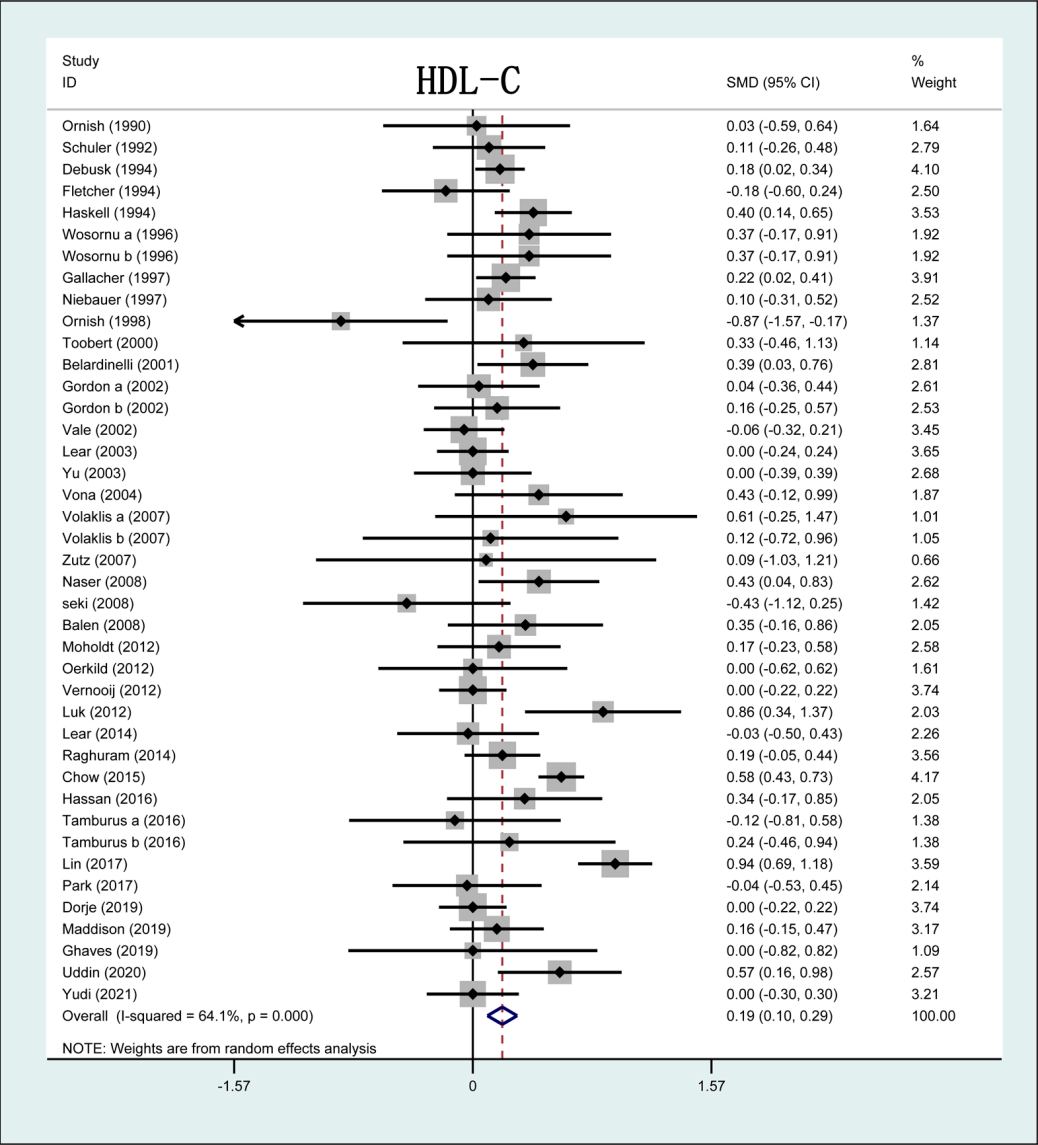
Forrest plot of HDL-C levels after CR treatment. HDL-C: high-density lipoprotein cholesterol; SMD: standardized mean difference.

Thirty-seven out of 51 studies included in this analysis reported on HDL-C levels after CR. The forest plot showed that the HDL-C serum levels in three studies were significantly higher in the CR group than in the control group. The results of the overall study revealed HDL-C serum levels were remarkably increased after CR (SMD = 0.19; 95%CI: [0.10, 0.29]; P < 0.001, Figure 3).

#### LDL-C levels

**Figure 4:**
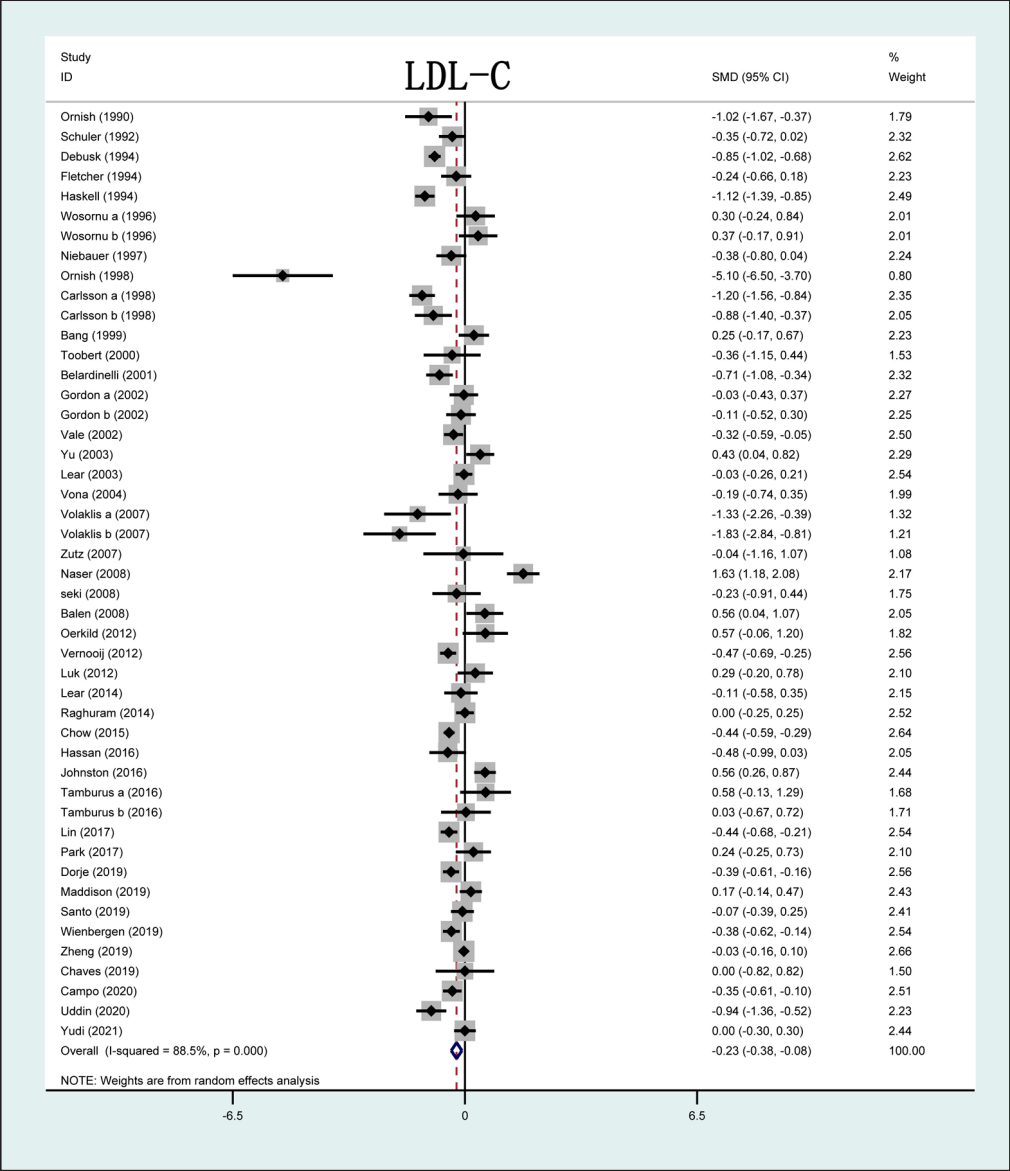
Forrest plot of LDL-C levels after CR treatment. LDL-C: low-density lipoprotein cholesterol; SMD: standardized mean difference.

Out of 51 studies, 42 included in this analysis reported on LDL-C levels after CR. The forest plot showed that the serum LDL-C levels in three studies were significantly lower in the CR group than in the control group. The results of the overall study revealed that compared with control group, LDL-C serum levels were reduced after CR (SMD = –0.23; 95%CI: [–0.38, –0.08]; P < 0.001, Figure 4).

#### TG levels

**Figure 5:**
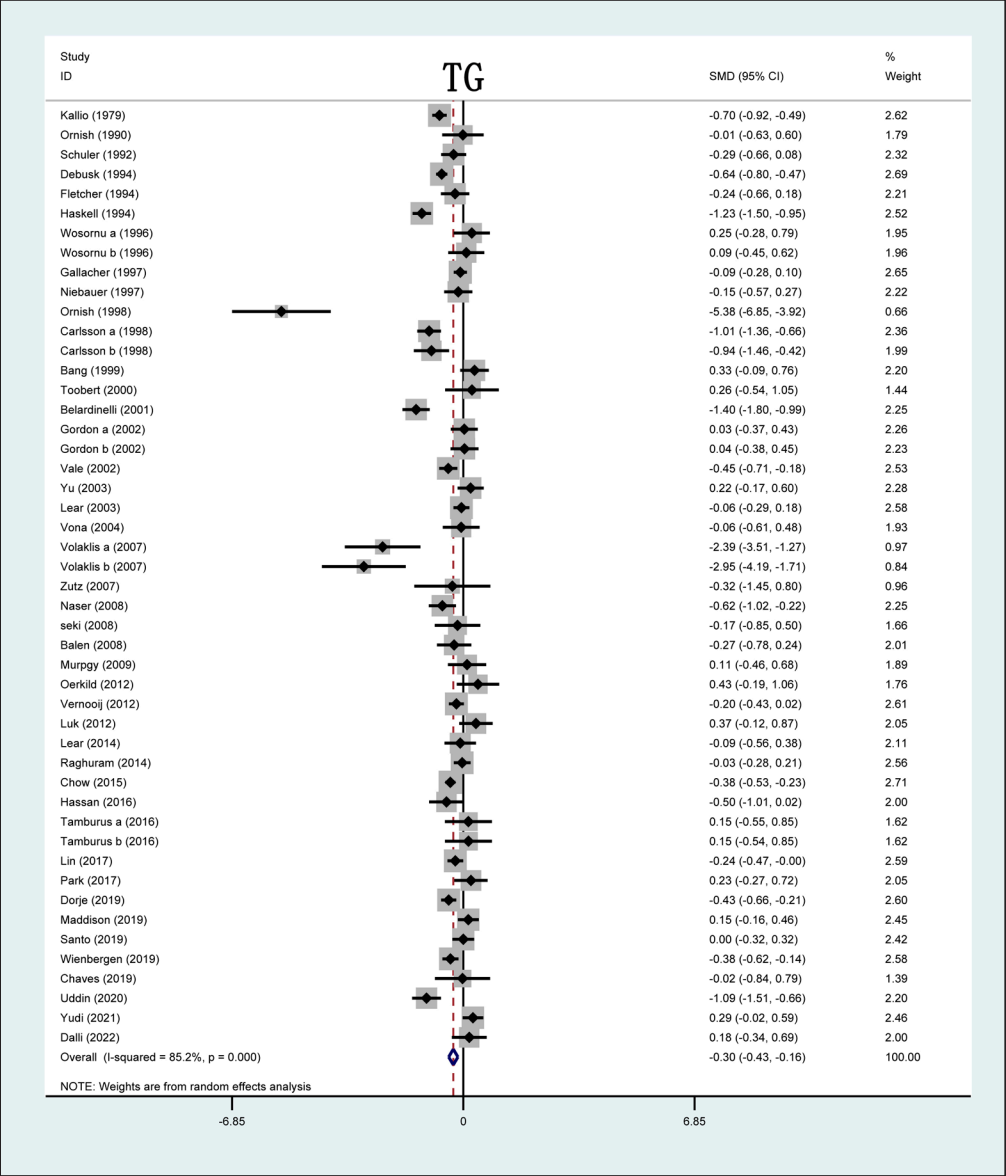
Forrest plot of TG levels after CR treatment. TG: triglyceride; SMD: standardized mean difference.

Out of 51 studies, 37 included in this analysis reported on TG levels after CR. The forest plot showed that TG serum levels in 10 studies were significantly lower in the CR group than in the control group. In the results of the overall study, significant decrease in serum TG levels were observed after CR (SMD = –0.17; 95%CI: [–0.28, –0.06]; P < 0.001, Figure 5).

#### Apolipoprotein A

Five out of 51 studies included in this analysis and reported on apolipoprotein A (ApoA) levels after CR. The forest plot of ApoA serum levels showed no significant differences between the CR and control groups (SMD = –0.46; 95%CI: [–1.22, 0.30]; P = 0.24, Figure S1).

#### Apolipoprotein B

Five out of 51 studies included in this analysis and reported on apolipoprotein B (ApoB) levels after CR. The forest plot of ApoB serum levels showed no significant differences between the CR and control groups (SMD = –0.94; 95%CI: [–2.01, 0.13]; P = 0.09, Figure S2).

### Subgroup analysis

The subgroup analysis was performed based on the following factors: publication year, sample size (<100 or ≥100), region, number of interventions, and study quality (score <7 or score ≥7).

### Subgroup analysis according to publication year

We executed a subgroup analysis based on publication year, and included studies were divided into three subgroups: before 2000, 2000–2010 and after 2010. CR could obviously increase HDL-C level in all three subgroups. However, CR significantly decreased LDL-C level in subgroups of before 2000 (SMD: –0.75; 95%CI: –1.12, –0.38) and after 2010 (SMD: –0.13; 95%CI: –0.38, –0.08). Remarkably reduced TC level in subgroups of before 2000 (SMD: –0.52; 95%CI: –0.81, –0.22) and 2000–2010 (SMD: –0.40; 95%CI: –0.70, –0.10) and lower TG level in subgroups of 2000–2010 (SMD: –0.29; 95%CI: –0.51, –0.06) and after 2010 (SMD: –0.23; 95%CI: –0.39, –0.08) were observed when compared with control group (Table S3–S6).

### Subgroup analysis according to sample size

Stratified group analysis was conducted based on the sample size and the studies were divided into two subgroups: those with number of patients less than 100 (<100) and greater than or equal to 100 (≥100). Results showed that in subgroup of sample size <100, LDL-C level was significantly decreased after CR (SMD: –0.32; 95%CI: –0.59, –0.04). In subgroup of sample size ≥100, TC (SMD: –0.39; 95%CI: –0.55, –0.24) and TG (SMD: –0.22; 95%CI: –0.34, –0.10) levels were considerably reduced after CR (Table S4–S6).

### Subgroup analysis according to the number of interventions

We also carried out the subgroup analysis in terms of number of interventions. Studies were divided into three subgroups: those with one type, two types and three types interventions. We found that HDL-C level was significantly improved in subgroup of one type intervention (SMD: 0.25; 95%CI: 0.11, 0.39) and two types interventions (SMD: 0.10; 95%CI: 0.02, 0.18) subgroup. LDL-C level was decreased in subgroup of two types interventions (SMD: –0.30; 95%CI: –0.49, –0.12). TC level was reduced in subgroup of one type (SMD: –0.32; 95%CI: –0.54, –0.11) and two types interventions (SMD: –0.30; 95%CI: –0.48, –0.13). TG levels were reduced in subgroup of one type (SMD: –0.20; 95%CI: –0.38, –0.02) and two types interventions (SMD: –0.12; 95%CI: –0.23, –0.01) (Table S3–S6).

### Subgroup analysis according to different regions

Subgroup analysis was performed in accordance with studies conducted in different regions. Based on the region, the studies were divided into six subgroups including US and Canada, Europe, Asia-Pacific, country combination, Africa, and South America subgroups. Based on the pooled results, we found that in US and Canada subgroup, LDL-C (SMD: –0.60; 95%CI: –0.95, –0.26) and TC (SMD: –0.49; 95%CI: –0.85, –0.14) levels were significantly reduced. In Europe subgroup, HDL-C (SMD: 0.17; 95%CI: 0.06, 0.28) was significantly improved, TC (SMD: –0.49; 95%CI: –0.85, –0.14) was reduced. In Asia-Pacific subgroup, HDL-C was increased (SMD: 0.24; 95%CI: 0.02, 0.45) and, TG level was reduced (SMD: –0.25; 95%CI: –0.41, –0.09). In combination subgroup, HDL-C was enhanced (SMD: 0.39; 95%CI: 0.03, 0.76) and, LDL-C (SMD: –0.71; 95%CI: –1.08, –0.34), TC (SMD: –1.4; 95%CI: –1.8, –0.99) and TG (SMD: –0.57; 95%CI: –0.94, –0.20) levels were all reduced. In Africa and South America subgroup, CR had insignificant effect on outcomes (Table S3–S6). As for ApoA and ApoB, we found insignificant differences between CR group and control group. We also did not find any potential sources of heterogeneity in this analysis (Table S1–S6).

### Publication bias

According to the results of Egger’s test and Begg’s funnel plots, there was no publication bias seen among TG, TC, HDL-C, LDL-C, ApoA, and ApoB. The details are presented in Table S7 and Figure S3.

## Discussion

This study highlights the effect of CR on lipid levels in patients with CHD. According to our results, CR can significantly reduce serum LDL-C, TC, and TG levels and increase serum HDL-C levels. The ApoA and ApoB levels did not change significantly.

The conclusion of our study is not in accordance with that of previous studies. In Chen’s study, LDL-C and HDL-C levels were improved after aerobic exercise, but TG and TC serum levels did not substantially change [8]. They collected four articles to conduct their analysis, though we have assembled 51 studies in our systematic review. The reason for the varied results may be the small number of articles on lipid analysis included in their studies. Similarly inconsistent with our conclusion, a meta-analysis by Javaherian et al. indicated that CR considerably reduced serum LDL-C, TC, and TG levels; however, no marked change was observed in HDL-C serum levels. They evaluated exercise-based CR and focused on all types of CR, including exercise, education, lifestyle modification, and so on. First, their CR range was more comprehensive. Second, our number of included articles was higher than that in their study. Third, they selected patients with cardiovascular disease (CVD) for analysis, while we restricted our review to patients with CHD. Few studies have focused on ApoA and ApoB; thus, the data were not sufficient.

In the subgroup analysis, we found that in one type and two types interventions subgroup, HDL-C levels were enhanced; LDL-C and TC levels were reduced. However, all endpoints found no significant differences in three types interventions subgroup; this might be due to inclusion of inadequate number of studies, (For each endpoint, only one study was included in three types interventions group). In subgroup of before 2000, LDL-C and TC levels were decreased. In subgroup of after 2010, LDL-C and TG levels were reduced. HDL-C was increased in all three subgroups according to the publication years. TC and TG levels were reduced in subgroup of sample size ≥100; LDL-C level was decreased after CR in studies with sample size<100. HDL-C, LDL-C, TG, and TC levels in group with study quality score higher than seven were obviously affected in positive way after CR. In terms of region, there were no magnificent differences observed in studies conducted in Africa and South America region after CR. Nevertheless, in other three regions, CR had remarkable effects on HDL-C, LDL-C, TC and TG. This difference might have been caused firstly by the inclusion of inadequate number of studies in Africa and South America region. Secondly, medical skills, types of insurance for commercially insured patients with different socioeconomic status and medication adherence were in different regions (Table S1-S6).

This study focused on comprehensively investigating the effects of CR on lipid profiles. In this study, CR was widely defined and not limited to specific types, such as exercise training. Moreover, all the trials included in this study were randomized controlled trials. Another strength of our study is the number of trials we were able to include in this review. Fifty-one articles provide a sufficient amount of trials for review. Consequently, our conclusions are hypothesized to be more reliable because of the larger number and higher quality of studies that we were able to incorporate in our systematic review.

### Limitations

Our analysis has some limitations. First, although the subgroup analysis was performed using a fixed model, the source of heterogeneity was still not observed. Second, in over 50 studies, most patients were male, and due to a lack of data assessing CR effect by sex, a subgroup analysis of sex was not performed. Third, we included articles only in English, and all other non-English and written literatures were excluded. Considering these limitations, the results should be interpreted with caution.

## Conclusion

In conclusion, this study illustrates that CR can reduce the levels of TC, TG, and LDL-C and improve levels of HDL-C. However, ApoA and ApoB levels did not change significantly after CR. Considering the efficacy of CR on improving blood lipid levels, which plays an important role in delaying disease progression and preventing recurrence and complications, CR should be widely promoted, and more clinical trials should be conducted regarding the long-term effects of CR.

## Data Accessibility Statement

All data generated or analyzed during this study are included in this published article and its supplementary information files.

## Additional Files

The additional files for this article can be found as follows:

10.5334/gh.1168.s1 Supplementary Figures. Figures S1 to S3.

10.5334/gh.1168.s2 Supplementary Tables. Tables S1 to S7.
