# Supplementary figures and images for: The Effect of Cardiac Rehabilitation on Lipid Levels in Patients with Coronary Heart Disease. A Systematic Review and Meta-Analysis
Source: Glob Heart. 2022 Nov 29;17(1):83. doi: 10.5334/gh.1170 (PMC9717003; doi:10.5334/gh.1170)

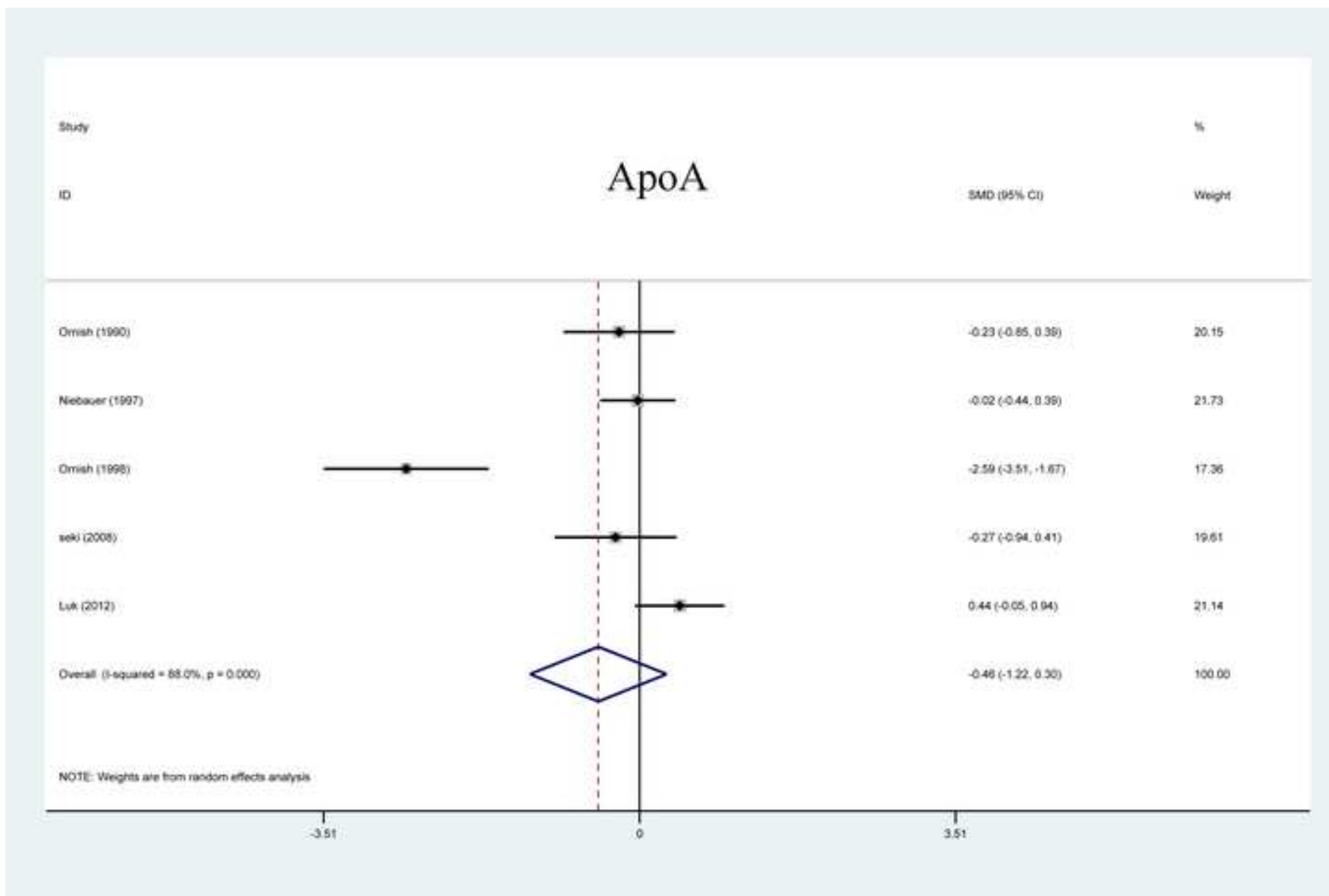

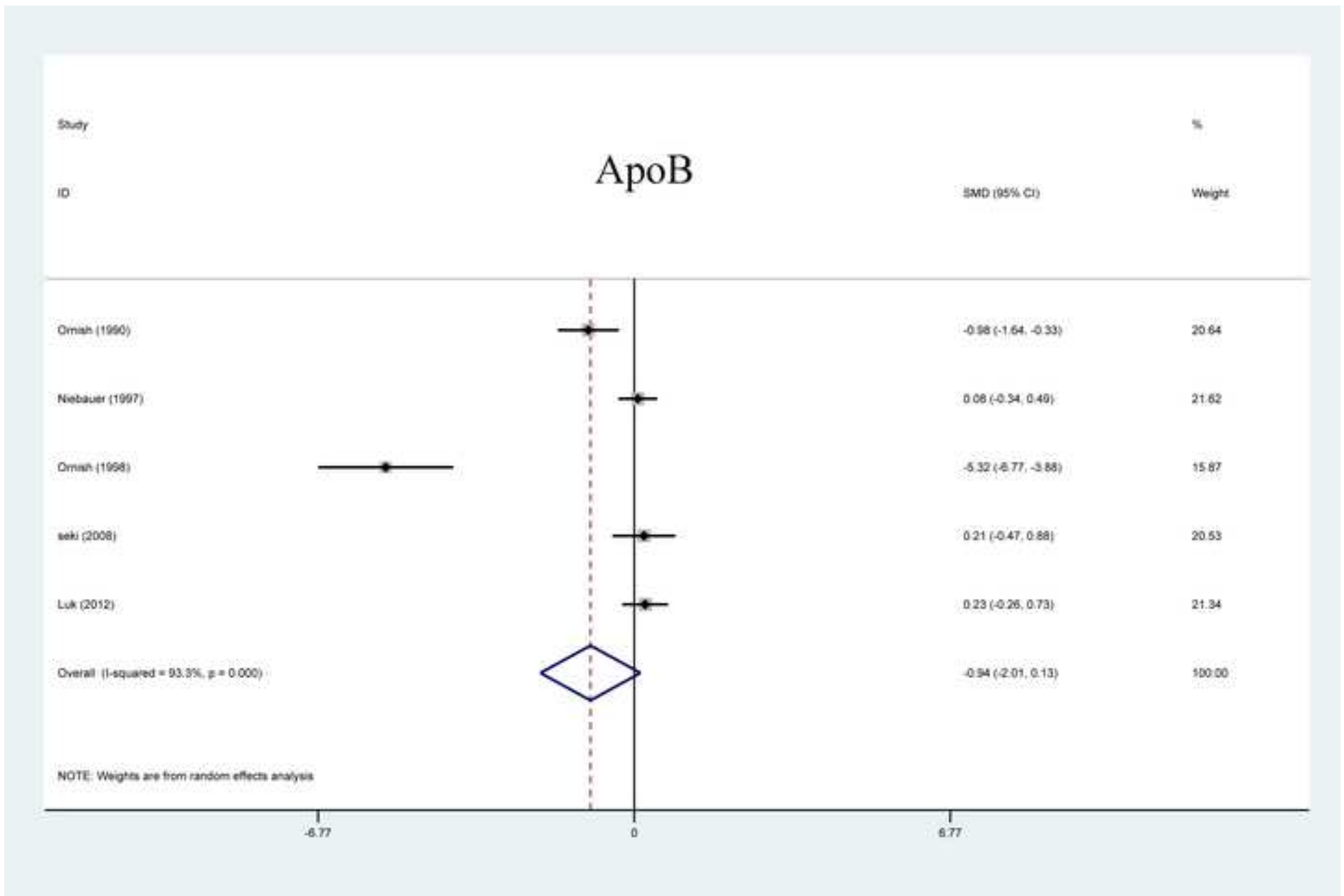

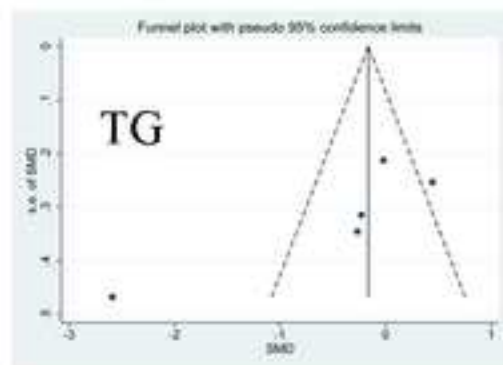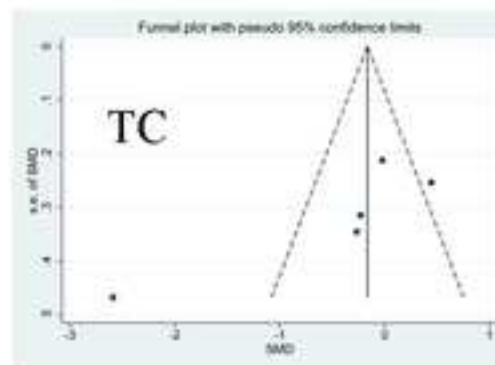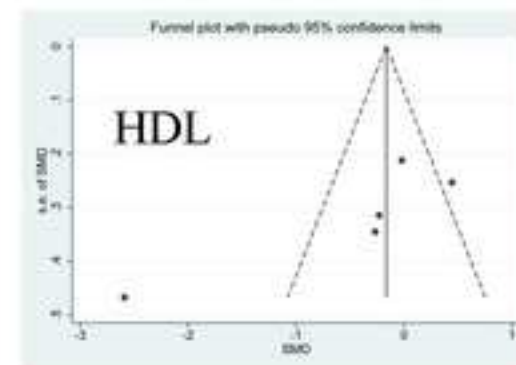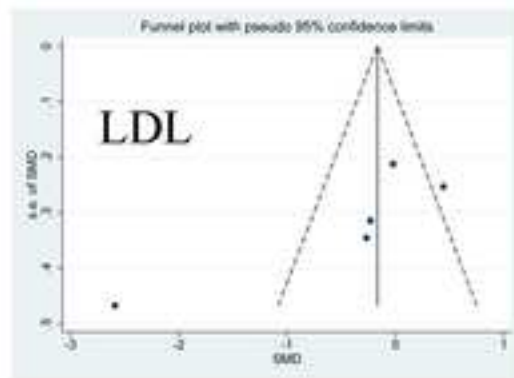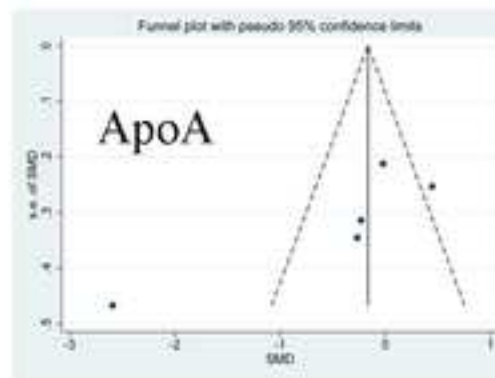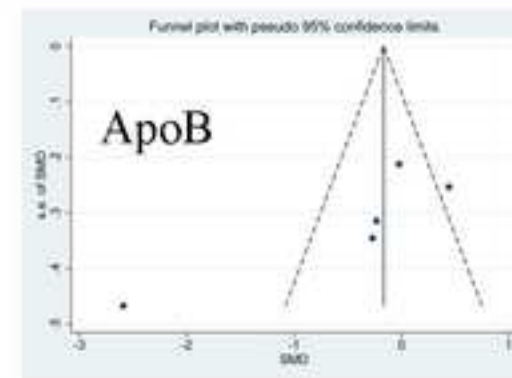

Supplement: Supplementary Figures. — Figures S1 to S3. [file gh-17-1-1170-s1.pdf]
