# Supplementary material for: The Effect of Cardiac Rehabilitation on Lipid Levels in Patients with Coronary Heart Disease. A Systematic Review and Meta-Analysis
Source: Glob Heart. 2022 Nov 29;17(1):83. doi: 10.5334/gh.1170 (PMC9717003; doi:10.5334/gh.1170)
Supplement: Supplementary Tables. — Tables S1 to S7. [file gh-17-1-1170-s2.pdf]

Table S1. Subgroup analysis of ApoA in heterogeneity

**Category**	No. of studies	Heterogeneity statistic	*P*	I^2	Tau^2
**Year**					
0 (before 2000)	3	25.46	0.00	92.1%	1.161
1 (2000-2010)	2	2.76	0.10	63.8%	0.162
**Region**					
0 (US and Canada)	2	17.58	0.00	80.6%	0.147
1 (Europe)	1	37.31	0.00	70.5%	0.097
2 (Asia-Pacific)	2	35.7	0.00	69.2%	0.047
**No. of intervention**					
0 (one type)	4	32.61	0.00	90.8%	1.081
1(two types)	1	0.00	0.00	NA	NA
**Quality of studies**					
1 (score ≥7)	5	33.32	0.00	88%	0.643
**Sample size**					
0 (<100)	4	32.61	0.00	90.8%	1.08
1 (≥100)	1	0.00	0.00	NA	0.00

Table S2. Subgroup analysis of ApoB in heterogeneity

Category	No. of studies	Heterogeneity statistic	*P*	I^2	Tau^2
**Year**					
0 (before 2000)	3	52.13	0.00	92.1%	1.16
1 (2000-2010)	2	0.00	0.95	0.00%	0.00
**Region**					
0 (US and Canada)	2	28.69	0.00	96.5%	9.09
1 (Europe)	1	0.00	0.00	NA	0.00
2 (Asia-Pacific)	2	0.00	0..95	0.00%	0.00
**No. of intervention**					
0 (one type)	4	56.99	0.00	94.7%	2.29
1(two types)	1	0.00	0.00	NA	0.00
**Quality of studies**					
1 (score ≥7)	5	57.96	0.00	93.3%	1.34
**Sample size**					
0 (<100)	4	56.99	0.00	94.7%	2.29
1 (≥100)	1	0.00	0.00	NA	0.00

Table S3. Subgroup analysis of HDL in heterogeneity

Subgroup	No. of studies	Heterogeneity statistic	*P*	I^2	Tau^2
**Year**					
0 (before 2000)	9	15.82	0.07	43.1%	0.02
1 (2000-2010)	12	13.97	0.38	6.9%	0.00
2 (2010-)	16	72.21	0.00	77.8%	0.09
**Region**					
0 (US and Canada)	12	20.28	0.06	40.8	0.03
1 (Europe)	10	6.43	0.84	0%	0
2 (Asia-Pacific)	12	76.54	0.00	74.9%	0.06
3 (combination)	1	0	0.00	NA	0.00
4 (Africa)	1	0	0.00	NA	0.00
5 (South America)	1	0.51	0.48	0	0
**No. of intervention**					
0 (one type)	23	80.07	0.00	68.8%	0.08
1(two types)	13	10.73	0.63	0%	0
2(three types)	1	0	0.00	NA	0
**Quality of studies**					
0 (score <7)	2	0.52	0.47	0%	0
1 (score ≥7)	35	103.66	0.00	63.3%	0.05
**Sample size**					
0 (<100)	18	27.76	0.15	24.4%	0.03
1 (≥100)	19	80.48	0.00	77.6%	0.06

Table S4. Subgroup analysis of LDL in heterogeneity

Subgroup	No. of studies	Heterogeneity statistic	*P*	I^2	Tau^2
**Year**					
0 (before 2000)	11	96.79	0.00	89.7%	0.32
1 (2000-2010)	13	101.75	0.00	86.2%	0.31
2 (2010-)	20	97.92	0.00	79.6%	0.80
**Region**					
0 (US and Canada)	12	112.52	0.00	89.3%	0.32
1 (Europe)	13	111.04	0.00	86.5%	0.25
2 (Asia-Pacific)	14	109.14	0.00	88.1%	0.13
3 (combination)	1	0.00	0.00	NA	0.00
4 (Africa)	1	0.00	0.00	NA	0.00
5 (South America)	1	1.18	0.28	15%	0.02
**No. of intervention**					
0 (one type)	23	244.73	0.00	89.8%	0.34
1(two types)	18	152.02	0.00	87.5%	0.15
2(three types)	1	0	0.00	NA	0.00
**Quality of studies**					
0 (score <7)	2	5.26	0.02	81%	0.18
1 (score ≥7)	40	388.03	0.00	88.7%	0.21
**Sample size**					
0 (<100)	21	127.03	0.00	81.1%	0.35
1 (≥100)	21	268.44	0.00	92.2%	0.19

Table S5. Subgroup analysis of total cholesterol in heterogeneity

**Subgroup**	No. of studies	Heterogeneity statistic	*P*	I^2	Tau^2
**Year**					
0 (before 2000)	12	139.28	0.00	90.7%	0.27
1 (2000-2010)	13	83.77	0.00	83.3%	0.26
2 (2010-)	18	64.51	0.00	72.1%	0.07
**Region**					
0 (US and Canada)	12	118.67	0.00	89.9%	0.33
1 (Europe)	15	98.62	0.00	82.8%	0.17
2 (Asia-Pacific)	13	47.87	0.00	74.9%	0.06
3 (combination)	1	0.00	0.00	NA	0.00
4 (Africa)	1	0.00	0.00	NA	0.00
5 (South America)	1	0.00	0.99	0.00	0.00
**No. of intervention**					
0 (one type)	24	209.23	0.00	87.6%	0.25
1(two types)	18	102.41	0.00	81.4%	0.11
2(three types)	1	0.00	0.00	NA	0.00
**Quality of studies**					
0 (score <7)	3	17.84	0.00	88.8%	0.23
1 (score ≥7)	40	298.02	0.00	85.2%	0.18
**Sample size**					
0 (<100)	20	106.91	0.00	78.5%	0.31
1 (≥100)	23	184.75	0.00	87.6%	0.13

Table S6. Subgroup analysis of Triglycerides in heterogeneity

Category	No. of studies	Heterogeneity statistic	*P*	I^2	Tau^2
**Year**					
0 (before 2000)	9	49.48	0.00	81.8%	0.13
1 (2000-2010)	12	42.06	0.00	69.1%	0.11
2 (2010-)	15	49.35	0.00	69.6%	0.06
**Region**					
0 (US and Canada)	11	56.65	0.00	80.6%	0.15
1 (Europe)	10	37.31	0.00	70.5%	0.10
2 (Asia-Pacific)	12	35.7	0.00	69.2%	0.05
3 (combination)	1	0.00	0.00	NA	0.00
4 (Africa)	1	0.00	0.00	NA	0.00
5 (South America)	1	0.91	0.34	0%	0.00
**No. of intervention**					
0 (one type)	22	113.35	0.00	78.8%	0.14
1(two types)	13	23.73	0.00	87.5%	0.15
2 (three types)	1	0.00	0.00	NA	0.00
**Quality of studies**					
0 (score <7)	2	0.59	0.44	0%	0.00
1 (score ≥7)	34	146.43	0.00	74.7%	0.10
**Sample size**					
0 (<100)	18	81.49	0.00	74.2%	0.24
1 (≥100)	18	64.31	0.00	73.6%	0.05

Table S7

Table S7.

	Begg's p	Egger's p (95% CI)
cholesterol	0.70	0.86 (-1.53, 1.83)
Triglycerides	0.94	0.42 (-0.80, 1.88)
HDL	0.56	0.18 (-1.93, 0.36)
LDL	0.70	0.63 (-1.41, 2.03)
ApoA	0.09	0.10 (-19.32,2.94)
ApoB	0.22	0.05 (-18.17,0.09)

Analysis of publication bias in all including studies
